# An International Comparative Study on the Resilience of Urban Communities after COVID-19 Pandemic: A One-Year Case Study between Lanzhou, China and Sarajevo, Bosnia and Herzegovina

**DOI:** 10.3390/ijerph192114458

**Published:** 2022-11-04

**Authors:** Dingwei Niu, Lucang Wang, Wei Li, Yongchi Ma

**Affiliations:** 1 College of Geography and Environmental Science, Northwest Normal University, Lanzhou 730070, China; 2 Gansu Guancheng Planning Design Research Co., Ltd., Lanzhou 730070, China

**Keywords:** community resilience, COVID-19, comparative study, Sarajevo

## Abstract

After the prevailing of the COVID-19 pandemic, urban communities around the world took initiatives to bring their cities back to life. In this research, 45 indicators and 55 elements were selected to make comparisons between urban communities in Lanzhou, China and Sarajevo, Bosnia and Herzegovina from five dimensions of social resilience, economic resilience, institutional resilience, infrastructural resilience, and community capital resilience. At the same time, the ArcGIS platform tool was used for spatial interpolation analysis. In this paper, the inverse distance weighting (IDW) method was used to carry out the spatial analysis of the perceived resilience of the two cities. Due to the heterogeneity of the neighborhood physical environment, operation and management mode, individual attribute characteristics, and internal relations, the resilience of the two urban communities showed disparity in different dimensions. Overall, the communities with good urban property management services, high-income owners, and the convenient transportation have stronger resilience in the face of pandemic. On the contrary, scattered communities, which are scattered in the inner cities, lack effective management, and based on unstable employment, people become the most affected by the epidemic with the lowest resilience power. The importance of social capital, represented by community understanding, identity, and mutual help and cooperation between neighbors, is highlighted in the resilience assessment of the two cities, respectively, in the East and West, indicating that to build more resilient cities, in addition to improving government management and increasing investment in urban infrastructure, building the residents’ sense of belonging, identity, and enduring community culture is even more important in the construction of resilient cities.

## 1. Introduction

The outbreak of the COVID-19 pandemic had a dramatic impact on the development of countries around the world. As of July 2022, the cumulative number of confirmed cases in the world exceeded 550 million, resulting in more than 6.356 million deaths [1]. Due to the severe infectious virus and the associated health risks, a series of changes were made on the development of countries over the world [2], urging different countries to adopt a series of different countermeasures, such as: restricting the flow of people, closing the port and airport [3], social distancing, placing restrictions on the import and export [4], taking the COVID-19 vaccine, etc. As different countries adopt different coping measures, the resulting impact on society’s economic development and on the psychological perception of people in the specific region are also different. Especially for rural communities near cities, this difference could be more apparent because it is more difficult to get the prevention and control information, vaccines, social assistance, etc., to rural communities near cities than the urban communities. More importantly, compared with people of urban communities, the difference in education, opinions, and the understanding of the coronavirus, cause people living in rural communities near cities to be at a disadvantage. The World Health Organization Regional Office for Europe always considered building community resilience and its supportive environment to be a public health priority [5], because this is directly related to what countermeasures we should take in the future, in the face of all kinds of unknown virus epidemics.

The word “Resilience”, mainly originated from physics, refers to the resilience of an object, that is, the reaction of a material to its external forces under the premise of maintaining its basic characteristics [6]. The core of the concept of resilience is the ability of things to return to their original and stable state after being destroyed or changed. Sajko et al. described resilience as the ability to predict, avoid, and adapt to sudden crises or shocks [7]. Creating resilient communities is not about leaving them alone to cope with shocks and their consequences, but about enabling public health systems to do some strengthening and protecting, such as building strong social networks that help people manage, adapt, and revitalize society [8]. In the face of the shock of COVID-19, J. South et al. proposed 11 elements for rebuilding community resilience based on social practice in the UK [9]. Gautam and Hens pointed out that COVID-19 had a greater impact on communities’ sustainability in emerging market economies, such as India and Brazil [10,11]. Siddharth Shankar Rai et al. incorporated three-dimensional models of society, economy, and environment into the study of community resilience, and combined with the sustainability of the community, emphasized that the community should maintain the sustainability of development while recovering from the crisis [12]. In the form of an online questionnaire, Shaoting Yue analyzed the cognition, attitude and countermeasures of urban and rural residents towards COVID-19, and pointed out that the inadequate cognition of rural residents towards the COVID-19 epidemic is a major obstacle to community health recovery [13]. There are usually two methods to measure communities’ resilience, namely based on objective indicators or subjective perception. Based on objective indicators, different studies discussed from the aspects of the vulnerability and coping capacity of resilience [14], the adaptability of resilience towards institutional economy and ecology [15], and the impact of space and time on communities’ resilience [16]. Studies based on subjective perception mainly focus on residents’ understanding of crisis and their initiative [17], rural residents’ empowerment, resistance [18], and perceived level of resilience [19]. The measurement method based on objective indicators mainly uses the statistical data offered by relevant departments of the country to extract and calculate the relevant index data of resilience, whose advantage is suitable for urban and above research areas, but its disadvantage is that it is difficult to obtain data from small research areas. However, method based on subjective perception can better restore the feelings of community residents and better reflect the role and significance of people in research. Current measurements mainly focus on social, economic, and ecological dimensions, and there is no unified standard in research methods. The qualitative and quantitative analysis methods for determining the weights of different dimensions include literature review, public participation, expert consultation, analytic hierarchy process (AHP), and entropy weight method of objective weighting.

Lanzhou and Sarajevo are located in East Asia and southeast Europe, respectively. They have many similarities, for example, they are both valley basin cities that are multi-ethnicity and multi-cultural. They both suffer from serious air pollution in the winter, and both face the threat of population aging. Take 2020 as an example; the per capita GDP of Lanzhou city and Sarajevo city is also relatively close, which is USD 11,039 and USD 11,000, respectively.

In this study, we focused on the following scientific issues:

(1) What are the differences in the perceived resilience of urban communities between Eastern and Western cities with similar development levels after taking different measures to deal with COVID-19?
(2) Within the city, faced with COVID-19, what are the differences of perceived resilience of different types of communities in five dimensions: social resilience, economic resilience, institutional resilience, infrastructure resilience, and social capital resilience?
(3) By analyzing the distribution of community perceived resilience in Lanzhou and Sarajevo, this paper tries to summarize the general law of community perceived resilience distribution in valley basin cities, and puts forward some suggestions for strengthening the construction of urban community resilience in the future.

We also focused on how community residents with different coping measures and cultural backgrounds perceive “resilience” in the context of COVID-19. By measuring the subjective perceived resilience of urban community residents in Lanzhou, China and Sarajevo, Bosnia and Herzegovina, we further probe how to build more “resilient” urban communities.

## 2. Materials and Methods

### 2.1. Study Area

Lanzhou City is located at 35°34′–37°07′ N and 102°36′–104°34′ E, in the transition zone from the Qinghai–Tibet Plateau to the Loess Plateau. With an altitude of 1520 m, Lanzhou is located in the geometric center of the land distribution of China, playing the role of an important central city, industrial base, and integrated transportation hub in northwest China. Lanzhou is a valley basin city, with the Yellow River running through. The urban area is of 1631.6 square kilometers, and the city’s permanent population is of 4,359,400 people. By July 2022, there were a total of 860 confirmed COVID-19 cases, 822 recovery cases, and 2 cases of death in Lanzhou [20]. The routes of COVID-19 epidemic transmission in Lanzhou mainly included family to family, within family, between cities (imported cases), within cities (spread cases), and in public places. Since the outbreak, Lanzhou took measures, such as restricting travel, wearing masks in public places, keeping social distancing, closing public places, and getting the vaccination.

Sarajevo City, which is 500 m above sea level, is located at 43°52′ N 18°25′ E in the Miljacka River valley, surrounded by Dinaric Mountains. Sarajevo, located in the geometric center of the triangular territory of Bosnia and Herzegovina, is the capital, political, economic, and cultural center of Bosnia and Herzegovina. The city Sarajevo is made up of four towns: Centar Sarajevo, Novi Grad Sarajevo, Novo Sarajevo, and Stari Grad Sarajevo. Sarajevo covers an area of 141.5 km^2^ and has a population of over 275,524 (according to the data in 2013). By March 2022, there were a total of 105,545 confirmed COVID-19 cases, 100,717 recovery cases, and 4828 cases of death in Sarajevo Canton, mainly in Sarajevo city [21]. Since the outbreak of COVID-19, the city adopted curfews, kept social distancing, closed public places, and obtained the vaccination.

### 2.2. Data and Research Methods

#### 2.2.1. Map Data

The basic geographic information data used in this study include digital elevation model data (DEM) and digital line graphic data (DLG). DEM data were provided by Geospatial Data Cloud site, Computer Network Information Center, Chinese Academy of Sciences. (http://www.gscloud.cn; accessed on 10 June 2022). The wired data comes from the network map platform OpenStreetMap (OSM). Point of interest (POI) data were obtained from Baidu map, and the coordinates were corrected.

#### 2.2.2. Questionnaire Data

The acquisition of questionnaire data can be divided into two stages. The pre-survey stage: In September 2021, the initial questionnaire was designed, and the urban apartment community, mountain slope courtyard community, suburban community, and urban scattered community were selected as sample spots for preliminary interview and questionnaire survey. Based on this, the questionnaire was revised and improved later. According to the improved questionnaire, research was conducted, respectively, in the main urban areas of Lanzhou city and Sarajevo city for about two weeks from October to November 2021. This research adopted the form of the combination of semi-structured interview and questionnaire distribution. A total of 935 valid questionnaires were received, including 556 in Lanzhou and 379 in Sarajevo.

### 2.3. Case Community Selection

#### 2.3.1. Community Classification and Definition

According to the actual situation of the two cities, in this research, based on the building and population organization form of the community, the urban community can be divided into urban apartment community, mountain slope courtyard community, suburban community, and urban scattered community. They are specifically defined as:

(1) Urban apartment community: It mainly refers to the community consisting of one or several adjacent buildings built by the same developer in the river valley basin. A large number of people live in these buildings, which have more than three stories. They generally have centralized water supply, power supply, and heat supply. There is specialized or part-time property management, and each floor has at least two sets of independent rooms.
(2) Mountain slope courtyard community: It mainly refers to the community composed of adjacent single-family low-rise buildings located on the hillside and mountain flat. These buildings are independently constructed by the residents, and each house usually has no more than three floors. They have independent gates, courtyards, and centralized water and power supply systems. However, the heating system is relatively independent, and the owner is responsible for the cleaning and property management of the house by himself.
(3) Suburban community: It mainly refers to the community composed of low-rise buildings close to each other in the suburbs of the city. These buildings are independently constructed by the residents, and there are no more than two stories in each house. The courtyard area is large. Generally, these houses have a centralized power supply and water supply system, but the residents heat the house themselves. The building is adjacent to farm fields, often with special storage rooms for farm tools.

Urban scattered community: This mainly refers to the community consisting of multiple independent buildings scattered within the city and adjacent to each other or broken and old multi-story buildings without property management. These buildings often lack unified planning, with poor supply of water and electricity, having no or few independent heating systems, and no independent courtyards.

#### 2.3.2. Community Selection

**Figure 1:**
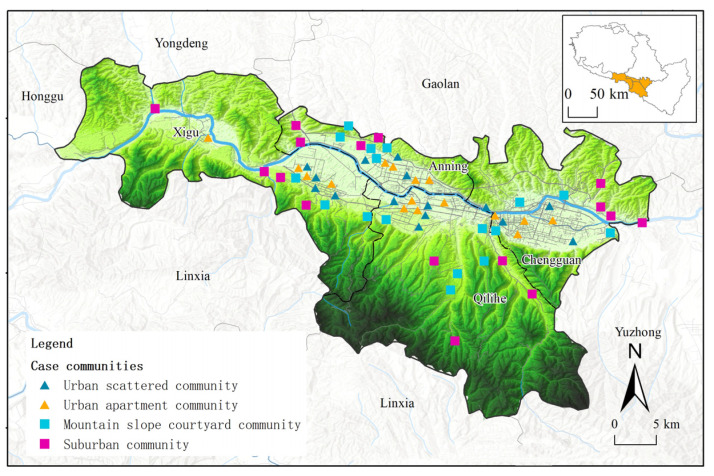
Case community distribution map of Lanzhou.

**Figure 2:**
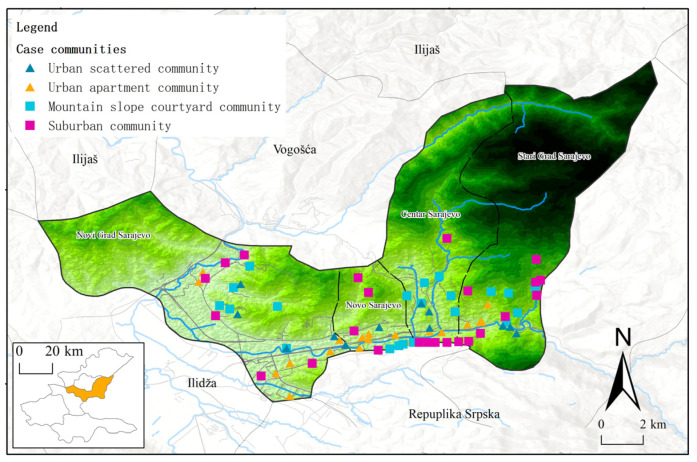
Case community distribution map of Sarajevo.

In accordance with the requirements of the local government, these case communities are all included in the zones where local epidemic control and management measures can be carried out, the same as other communities in the area, to implement the epidemic prevention and control requirements. In Lanzhou, the typical communities in the main urban areas of the city, namely Chengguan District, Qilihe District, Anning District, and Xigu District, were selected for investigation (Figure 1, Table A1). In regard to Sarajevo city, we selected typical communities in Centar Sarajevo, Novi Grad Sarajevo, Novo Sarajevo, and Stari Grad Sarajevo to investigate (Figure 2, Table A2). In each district, in order to ensure the comparability, comprehensiveness and validity of the samples, 4–8 communities of different types were selected in each district of the urban space.

### 2.4. Research Methods

#### 2.4.1. Model Construction

Quantitative evaluation of urban resilience mainly includes process-based evaluation and state-based evaluation.

(1) When it is described as a process, the measurement of resilience mainly focuses on the continuous change and recovery of system functions and the numerical level of resilience. Usually, it is characterized by the whole process of continuous and dynamic change in a certain functional index with time before and after a disaster. The specific method is to construct the function of resilience level with time, including system performance curve and production function. Process-based evaluation and measurement of urban resilience mostly appear in specialized research fields, especially in the research of infrastructure resilience and economic resilience. It mainly focuses on the degree of damage and recovery of specific infrastructure after disasters, the state of macroeconomic recession and recovery, and usually uses time series data to fit its changing characteristics [22].

(2) When described as a result state, the measure of resilience mainly focuses on the ability of the system function to keep running. There are two common ways to characterize this ability: ① Measure the recovery time and the degree of recovery and reconstruction due to disaster losses, and quantitatively describe its recovery ability. ② Quantitative evaluation with the help of an index system to characterize and statically describe the anti-disturbance, recovery and adaptability of the system. As this method is regarded as an inherent state and characteristic of the system, it can be regarded as a benchmark for providing resilience construction, so the resilience measured under this path is usually called “baseline resilience”. State-based resilience evaluation and measurement is especially suitable for urban and rural planning and disaster management, and provides comprehensive risk management strategies and urban construction standards for improving disaster prevention capabilities [23]; it provides quantitative support for the comparison of spatial heterogeneity and development differences among different cities, communities, land use types and aid recipients [24,25,26], and provides reference for the adjustment of relevant urban policies after the disaster.

As this study focuses on the comparison between Eastern and Western cities and communities, with the aim of further enhancing the ability of cities to resist disasters and providing comprehensive risk management strategies for urban construction, it draws on the Baseline Resilience Indicators for Communities (BRIC) proposed by Cutter, etc. [27]. The BRIC originated from a study by American scholars [28]. It is the representative of the index system evaluation method under the state-based toughness evaluation path. It identifies 49 indicators as individual evaluation factors from six aspects of society, economy, system, infrastructure, ecology, and social functions, as well as analyzes and measures the community resilience level of 3108 counties in the United States, and discusses the minimum construction standards that should be met in the process of improving urban resilience [23].

This study verifies and improves the measurement tools in combination with the disturbance of the epidemic disasters on urban communities and the characteristics of the case communities themselves, and constructs a measurement model of community resilience under public health emergencies. The model is composed of five dimensions: social resilience, economic resilience, institutional resilience, infrastructural resilience, and community capital resilience. Dimensions complement each other to comprehensively characterize the resilience of urban communities facing pandemic disasters. At the same time, the secondary and tertiary indicators under each measurement dimension were determined to build the indicator system, aiming to quantitatively measure the community’s ability to cope with public health emergencies.

#### 2.4.2. Indicators Selection

Cutter suggested that the community should seek open and transparent resilience indicators that are consistent with the community’s goals and vision. At the same time, he pointed out that the community resilience index needs to be simple and well documented, able to cope with multiple disasters, replicable by others, able to describe the geographical scope, physical dimensions, and community members, and possess certain extensibility to flexibly adapt to different communities and environmental changes. From the community’s point of view, an effective community resilience index should represent whether the community has the ability to respond to and recover from disasters accurately, reliably, comprehensively, expansively, affordably, and executively. BRIC adds resilience factors of social and organizational systems to measure the effectiveness of projects, policies, and interventions to improve disaster resilience. BRIC includes 36 indicators in five dimensions: social resilience, economic resilience, institutional resilience, infrastructure resilience, and community capital resilience. Through BRIC, we can monitor the change in resilience of a specific place with time, and compare the characteristics of the social resilience of two cases.

Based on the existing research results, combined with the disturbance and prevention and control characteristics of the epidemic outbreak, the indicators related to community epidemic prevention and control, as well as residents’ anti-disaster ability were selected from five measurement dimensions, and then the corresponding description elements for each indicator were selected to construct an evaluation system. By exploring the correlation between various factors and eliminating highly correlated variables, 45 indicators and 55 description elements were finally obtained (Table 1).

(1) Social resilience

Social resilience is the ability of a community to absorb, recover, and improve as reflected by the individual characteristics of its residents when the community is disturbed by the pandemic. It was measured by age structure, education structure, time of residence, health status, medical insurance, information skills, risk awareness, proportion of the elderly population in the community, and so on.

(2) Economic resilience

As the key part of a community response to an emergent public health event, economic resilience not only provides financial security for the community and its residents against epidemic disaster, but also will help ease residents’ unemployment panic caused by the community management and control under the sudden outbreak of an epidemic. Economic resilience mainly reflects residents’ ability to maintain a normal life and ensure a certain income during the outbreak. Through occupation, income, house asset, and other indicators, community residents’ economic resilience can be reflected.

(3) Institutional resilience

Institutional resilience is the focus of a community response to public health emergencies. The effectiveness and perfection of institution and management determine the ability to fight against epidemic disasters and the completeness of community in the whole process of prevention and warning, disaster assessment and response, and post-disaster recovery. The institutional resilience of the community was measured through indicators such as emergency prevention and control, policy coverage, disaster prevention awareness, prevention and early-warning construction, organizational leadership ability, and community work improvement.

(4) Infrastructural resilience

Infrastructural resilience, as the basis of community resilience in dealing with public health emergencies, mainly reflects the rationality, accessibility, and completeness of the distribution of various infrastructure and public service resources around the community. It was measured by medical facilities, life supporting facilities, leisure space, material supply, the convenience level of transportation, emergency placement places, landscape greening, and other indicators.

(5) Community capital resilience

As the core of public health emergency response, community capital resilience mainly reflects the cohesion and coordination between community residents and community management levels to cope with the epidemic, as well as the accessibility of information and resources. Community participation, identity, social network relationship, collective memory, and other indicators were selected for this measurement.

#### 2.4.3. Data Analysis Methods

(1) This section Reliability and validity analysis

By using SPSS 23.0 statistical analysis software to examine the reliability and validity of the questionnaire, the results show that Cronbach’s α reliability coefficient was 0.722, KMO = 0.877, and the Bartlett test *p* = 0, indicating that the questionnaire had good reliability and validity. The characteristics of the respondents are authentic and valid.

(2) Data normalization

In order to avoid the influence of the original data on the comprehensive measurement result due to the difference in indicator property, order of magnitude, and dimension of quantity, the original data were first normalized before data analysis. In this paper, the original data are divided into positive and negative indicators by a min–max normalization method. The specific methods are as follows:

Positive indicator: the larger the indicator value, the more favorable it is to the superior target. The normalized formula of the positive indicator is adopted:

(1) $$Y_{ij}=(X_{ij}-X_{j\min})/(X_{j\max}-X_{j\min})$$

Negative indicator: the larger the indicator value, the more unfavorable it is to the superior target. The normalized formula of the negative indicator is adopted:

(2) $$Y_{i}{}_{j}=(X_{j\max}-X_{ij})/(X_{j\max}-X_{j\min})$$

In the formula: $Y_{ij}$, $X_{ij}$, $X_{j\max}$, $X_{j\min}$ are, respectively, the normalized value, original value, maximum value, and minimum value of the number j indicator of the number i evaluation object. i=1,2,⋅⋅⋅,m; j=1,2,⋅⋅⋅,n.

(3) Entropy weight TOPSIS

Entropy weight method is an objective weight method, which can calculate the entropy weight of each index by using information entropy according to the degree of variation in each index, and then modify the weight of each index by entropy weight, so as to obtain a relatively objective weight of the index. The technique for order preference by similarity to ideal solution (TOPSIS) is a multi-objective decision making method, which is often used in multi-index comprehensive evaluation. However, it is necessary to determine the index weight in advance to avoid the randomness of the results. The advantages of the two methods are integrated, so the improved TOPSIS method can effectively eliminate the influence of the subjective weight of the traditional TOPSIS method, and at the same time, it can objectively reflect the degree of variation in indicators. The advantage of this method is that it has no strict restrictions on data distribution, sample size, and number of indicators. It is suitable for small systems with small samples and few indicators. It is also suitable for large systems with large samples and many indexes. Moreover, its mathematical calculation process is moderate, and it is widely used in many fields, such as urban resilience evaluation [29,30], policy benefit evaluation [31,32], public health decision making [33], and so on.

By making the best and worst solutions for each indicator, the technique for order preference by similarity to ideal solution (TOPSIS), sorts the evaluation objects out through their closeness to the best and worst solutions, as the basis for evaluating the relative merits of the object. The specific calculation steps are as follows:

① Determine the indicator weight *W*. Use the entropy weight method to determine the weight of the community resilience evaluation indicator, and the calculation process is as follows:

(a) Based on normalized value *Y_ij_*, calculate *P_ij_*: the weight of the number evaluation object under the number *j* indicator.

(3) $$P_{ij}=Y_{ij}/\sum_{i=1}^{m}Y_{i}{}_{j}$$

(b) Calculate the entropy *e_j_* of number indicator:

(4) $$e_{j}=-K\sum_{i=1}^{m}P_{ij}Lnp_{ij},k=1/Lnm$$

(c) Calculate the coefficient of difference $g_{j}$ of number indicator:

(5) $$g_{j}=1-e_{j}.$$

(d) Calculate the weight $w_{j}$ of number indicator:

(6) $$Wj=g_{j}/\sum_{j=1}^{m}g_{j}$$

② Construct weighted normalized matrixing matrix *Z*:

(7) Z=Y∗W

③ Determine the optimal solution $Z^{+}$ and the worst solution $Z^{-}$:

(8) $$\begin{array}{l} Z^{+}=\left\{\max Z_{ij},j=1,2,\cdots ,n\right\}=\{Z_{1}^{+}+Z_{2}^{+}+\cdots +Z_{n}^{+}\}\\ Z^{-}=\left\{\max Z_{ij},j=1,2,\cdots ,n\right\}=\{Z_{1}^{-}+Z_{2}^{-}+\cdots +Z_{n}^{-}\} \end{array}$$

In this formula: $Z_{ij}=Y_{ij}*W_{j}$;

④ Calculate the Euclidean distance of each evaluation object to the optimal solution and to the worst solution:

(9) $$D_{i}^{+}=\sqrt{\sum_{j=1}^{n}{\left(Z_{ij}-Z_{j}^{+}\right)}^{2}};D_{i}^{-}=\sqrt{\sum_{j=1}^{n}{\left(Z_{ij}-Z_{j}^{-}\right)}^{2}}$$

⑤ Calculate the relative proximity of each evaluation object to the optimal solution $C_{i}$:

(10) $$C_{i}=\frac{D_{i}^{-}}{D_{i}^{+}+D_{i}^{-}}$$

In this formula: $C_{i}$ is community resilience index $0\leq C_{i}\leq 1$. A larger value of $C_{i}$ indicates that the sample is closer to the optimal solution, and the community resilience against public health hazards is stronger, and vice versa.

Of all the above formulas, i=1,2,⋅⋅⋅,m; j=1,2,⋅⋅⋅,n.

(4) Inverse Distance Weighting (IDW) method

In this study, the spatial interpolation analysis of the survey sample data and the simulation prediction are carried out to obtain the spatial distribution characteristics of the area. Based on the ArcGIS platform tool, the inverse distance weighting (IDW) method is used for spatial interpolation analysis. Things that are closer to each other are more similar than things that are farther apart. When making a prediction for any unmeasured location, the inverse distance weighting method takes measurement value around the predicted location. Measurements closest to the predicted location have a greater impact on the predicted value than measurements farther away from the predicted location. The inverse distance weighting method assumes that each measurement point has a local effect, which decreases as the distance increases. This method assigns more weight to the points closest to the predicted position, but the weight decreases as a function of distance. The calculation formula is as follows:

(11) $$f\left(x,y\right)=\frac{\sum_{i=1}^{n}\left(1/d_{i}^{k}\right)\cdot Z_{i}}{\sum_{i=1}^{n}1/d_{i}^{k}}$$

In this formula: f(x,y) is the predicted value at the coordinate point (x,y); $Z_{i}$ is the measured value at (x,y); n is the number of predicted points around sample points participating in interpolation; $d_{i}$ is the distance between the predicted points and each known sample point; and k is the specified power.

In this formula, *k* is also the weight, which is generally taken as 1–2, and 2 is usually taken in calculation.

## 3. Results and Analysis

### 3.1. Results

#### 3.1.1. Measurement Results of Perceived Resilience of Communities in Different Cities

Take the original data into the above formula to calculate the perceived resilience value of urban communities. Facing the COVID-19 outbreak, the results show:

For Lanzhou, China, the average urban community resilience index was 0.5157. In terms of the resilience index of different types of communities, urban apartment community was the highest (0.6606), followed by suburban community (0.5163), mountain slope courtyard community (0.4697), and urban scattered community was the lowest (0.4161). When the resilience value of different dimensions are comprehensively compared, the rankings are as follows: community capital resilience (0.5789) > social resilience (0.5333) > institutional resilience (0.5202) > economic resilience (0.4927) > infrastructural resilience (0.4534).

For Sarajevo, Bosnia and Herzegovina, the average urban community resilience index was 0.4533. In regard to the resilience index of different types of communities, mountain slope courtyard community was the highest (0.5263). It was followed by urban apartment community (0.4843) and suburban community (0.4419). The lowest was found in urban scattered communities (0.3831). Comprehensively compared, the resilience values of different dimensions are ranked as follows: community capital resilience (0.5564) > social resilience (0.5131) > institutional resilience (0.4195) > infrastructural resilience (0.3930) > economic resilience (0.3843).

#### 3.1.2. Measurement Results of Perceived Resilience of Different Types of Communities

Through the comparison of various communities, the differences in resilience of different types of communities in various dimensions and comprehensive resilience index can be seen. From the perspective of resilience in various dimensions of different types of communities:

(1) Urban apartment community. In terms of the five dimensions measuring community resilience, for the urban apartment community in Lanzhou, the social resilience (0.7191) is the highest, followed by the institutional resilience (0.7104), community capital resilience (0.6602), economic resilience (0.6140), and infrastructural resilience (0.5995). For the urban apartment community of Sarajevo, community capital resilience (0.5663) is the highest, followed by economic resilience (0.5183), social resilience (0.5136), infrastructural resilience (0.4714), and institutional resilience (0.4520).

(2) Mountain slope courtyard community. From the five dimensions of measuring community resilience, for the mountain slope courtyard community in Lanzhou, community capital resilience (0.6665) is the highest, followed by social resilience (0.4726), economic resilience (0.4659) and institutional resilience (0.4215), and infrastructural resilience (0.3221) is the lowest. As that of Sarajevo city, the community capital resilience (0.6106) is the highest, followed by social resilience (0.6039), institutional resilience (0.5315) and infrastructural resilience (0.4924), and economic resilience (0.3933) is the lowest.

(3) Suburban community. In terms of the five dimensions measuring community resilience, for the suburban community in Lanzhou, the community capital resilience (0.7238) is the highest, followed by social resilience (0.5700), institutional resilience (0.4620) and infrastructural resilience (0.4225), and economic resilience (0.4032) is the lowest. For Sarajevo’s suburban communities, community capital resilience (0.5836) is the highest, followed by economic resilience (0.5197), social resilience (0.4767) and institutional resilience (0.3973), and infrastructural resilience (0.2292) is also significantly lower.

(4) Urban scattered community. From the five dimensions of measuring community resilience, for the urban scattered community in Lanzhou, infrastructural resilience (0.5694) is the highest, followed by institutional resilience (0.4870), economic resilience (0.3879), social resilience (0.3713), and community capital resilience (0.2652) is the lowest. For Sarajevo’s urban scattered community, community capital resilience (0.4652) is the highest, followed by social resilience (0.4580), infrastructural resilience (0.3789), economic resilience (0.3160), and institutional resilience (0.2972) is the lowest.

#### 3.1.3. Distribution of Perceived Resilience in Different Urban Communities

**Figure 3:**
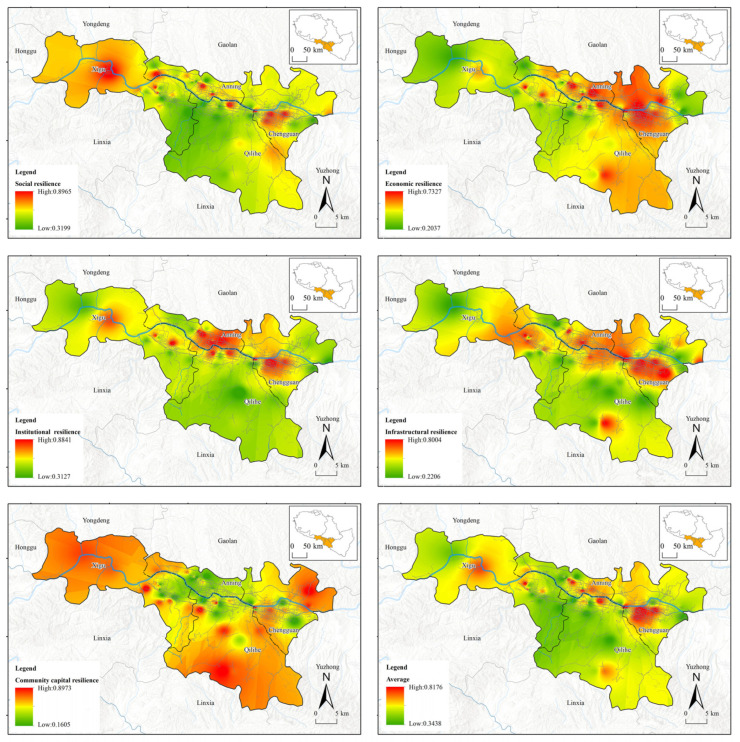
Distribution map of perceived resilience in different dimensions in Lanzhou.

**Figure 4:**
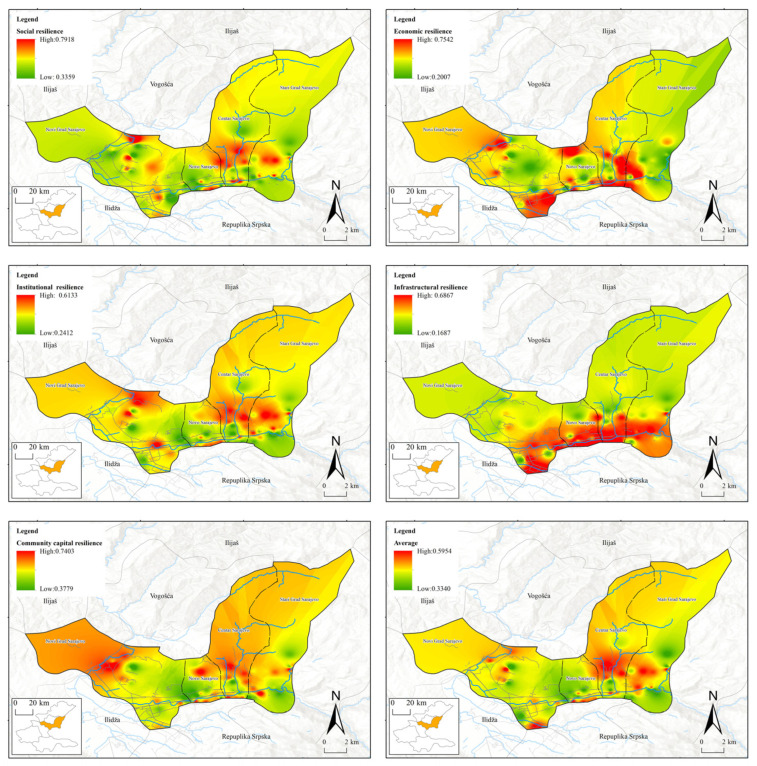
Distribution map of perceived resilience in different dimensions in Sarajevo.

Through interpolation analysis, this paper obtains the distribution maps of perceived resilience in different dimensions in Lanzhou (Figure 3) and Sarajevo (Figure 4). we found that, under the influence of COVID-19, the cities with river valleys and basins were with the following characteristics:

(1) The average perceived resilience value of the old towns is higher than that of the newer towns.

In Sarajevo, the perceived resilience of communities built from the Osman Ottoman Turkish era, and continuously developed through the Austro-Hungarian era and the socialist era under Tito, are stronger than those built after the war of the 1990s. The same situation came about in Lanzhou. Communities inhabited since the Ming and Qing Dynasties have a higher perceived resilience value than those newly built in recent years. The common features of these old towns are that inhabitants live there over generations with complete street systems, moderately sized and well-distributed shopping outlets, and perfect medical emergency facilities, though the buildings and streets in these neighborhoods are slightly older. However, the new commercial and residential areas in the city, even though they have vast lawns, squares, high-rise or super high-rise buildings, large shopping malls, as well as convenient subway and parking lots, do not have strong perceived resilience.

(2) The areas with high values of economic resilience and infrastructural resilience show obvious distribution characteristics along the river.

In Sarajevo, areas with high values of economic resilience and infrastructural resilience are clearly distributed along the Milijacika river. In those communities that are built along the river, residents have more stable employment and higher incomes, and they can enjoy trams, taxis, and self-driving cars built along the river. In Lanzhou, communities along the Yellow River also emerged as areas of high economic resilience and infrastructural resilience, with residents generally having higher incomes and more stable jobs than those in other regions. Public transportation, such as subways, buses, and taxis, are also more developed than those in other regions, and residents in these communities also have a stronger capability of resisting the risks brought by the epidemic.

(3) The areas with high values of community capital resilience show the characteristics of fleeing from the central urban area.

Community capital resilience, characterized by mutual understanding, recognition, help, and collaboration among community residents, shows the feature of fleeing from the central urban areas under the COVID-19 pandemic. The emerging communities in urban centers lack basic communication among residents, let alone mutual help and shared community memory or community spirit, and community capital resilience in these spaces is also becoming weaker in the face of COVID-19. On the contrary, there are some high-value areas of community capital resilience in urban suburbs and traditional mountain slope courtyard communities, where community residents have higher perceived resilience of community capital and never feel “alone” confronting COVID-19.

(4) Social resilience and institutional resilience highly fit each other in space.

Through the analysis, it can be found that the residents’ social resilience and institutional resilience highly fit each other in space. Community residents with a higher education level, longer urban residence period, higher overall satisfaction with society, and better use of smart phones and other information devices also have stronger institutional resilience under the pandemic, which can be highlighted by their ability to understand and cooperate with the government’s epidemic prevention and control measures. Public institutions in their communities also have a greater capacity for emergency support and smooth information transmission.

### 3.2. Analysis

#### 3.2.1. Urban Apartment Community

Compared with the two cities, the average resilience value of urban apartment communities in Lanzhou (0.6606) was higher than that in Sarajevo (0.4843). In terms of institutional resilience, as the urban apartment communities in Lanzhou are more scientific and effective in the implementation of epidemic prevention and control policies, potential patient screening, treatment of infected cases, and public material support, they show stronger government organization ability and mobilization ability, and their resilience value is relatively high. In terms of social resilience, the age structure of the elderly, middle-aged, and young residents in the apartment communities in Lanzhou was reasonable, and the education level, health level, and the levels of using information technology were relatively higher than those in Sarajevo, with the residents’ understanding of the epidemic being more scientific. In regard to economic resilience, due to bank loans, employment pressure, rising prices, and other reasons, residents in apartment communities in Lanzhou have an insufficient ability to withstand the attack of the epidemic, resulting in their low economic resilience, low overall income level, and low consumption level. However, residents in Sarajevo with sound social security systems basically were not interrupted. In terms of infrastructural resilience, although the overall infrastructure level in Lanzhou city is higher than that in Sarajevo, due to the strict control measures during the epidemic, Lanzhou’s relatively developed infrastructure did not play a proper role. Residents lack the perception for the effectiveness of the infrastructure, exposed the urban infrastructure’s disadvantage in distribution, service range, and the level of convenience. In Sarajevo, on the contrary, although the infrastructure was relatively poor, it worked well during the pandemic, especially the free movement of private cars, which effectively connected the city’s infrastructure.

#### 3.2.2. Mountain Slope Courtyard Community

Compared with the two cities, the average mountain slope courtyard community resilience in Sarajevo (0.5263) was higher than that in Lanzhou (0.4697). In terms of infrastructural resilience, people living in the mountain slope courtyard community in Sarajevo are less dependent on the public transportation system and have more robust community service facilities, with less impact on infrastructure services. In contrast, in Lanzhou, due to the lack of supporting medical treatment facilities and rest facilities in the mountain slope courtyard community, and residents highly depend on the public transportation system for external transportation; at the same time, with the government’s strict epidemic control measures, the residents’ perceived resilience decreased during the epidemic. A convenient, small-scale infrastructure that can fully meet the needs of the community population is of great significance for the construction of the resilient mountain slope courtyard community, and this is reflected in this study. In social resilience, Sarajevo’s mountain slope courtyard community is populated by members of the urban middle class and above, usually living together with several generations, and with regular occupations and high levels of education. However, those who live in the mountain slope courtyard community in Lanzhou are the indigenous people who lived for generations, or the “new immigrants” who have only come in recent decades or some tenants. Most of them do not have regular occupations, and education level is also not high. Most of them are families with the elderly left-behind, or young people with young children who are new to the city for work. These communities have lower social resilience than Sarajevo’s mountain slope courtyard community. As for institutional resilience, Sarajevo residents in the mountain slope courtyard community were well aware of the government’s outbreak management policies and were able to work with the government to implement effective governance and quickly return to normal conditions after COVID-19. However, in the mountain slope courtyard community in Lanzhou, due to the instability of employment and income, many people could not fully cooperate with the government’s epidemic prevention and control measures during the epidemic, forcing the government to make many policy changes in management, which was difficult to recover after the epidemic. As for community capital resilience, residents living in the mountain slope courtyard community in Lanzhou city, no matter “indigenous people” or “new immigrants”, are influenced by Chinese traditional culture, most of whom are very familiar with community neighbors, and some even have blood relationships. In this situation, the phenomena that relatives help relatives and friends help friends is common. What is more, they live together in a community. Residents’ community identity, as well as community support, is stronger. Although this situation also exists in Sarajevo, under the influence of Western culture, neighbors always abide by the characteristics of mutual respect, non-interference, and independence.

#### 3.2.3. Suburban Community

Compared with the two cities, the average resilience of suburban communities in Lanzhou (0.5163) was higher than that in Sarajevo (0.4419). In addition to economic resilience, the suburban communities in Lanzhou are significantly more resilient than those in Sarajevo in many dimensions. In terms of infrastructural resilience, the suburban communities in Lanzhou are equipped with health centers, villagers’ squares, primary and secondary school buildings and playgrounds for temporary shelters, and the road traffic conditions from the suburbs to the city center are also relatively perfect. Sarajevo clearly falls short in these areas. In regard to community capital resilience, most residents in the suburban communities in Lanzhou live in the family style and have strong blood ties with each other. The strong family spirit often becomes the value basis for neighbors to help each other and cooperate with each other, with strong social mobilization ability. In terms of economic resilience, Lanzhou is still in the process of rapid urbanization. A large number of unstable workers are concentrated in the suburbs of the city. They are either “indigenous people” working in the city or “migrant workers” from surrounding areas. In the face of the pandemic, the economy situation of residents of suburban communities is increasingly vulnerable to the falling incomes and rising unemployment, as well as strict government policies to contain the virus. On the contrary, the residents of Sarajevo’s suburban communities are more resilient to the pandemic due to the limited economic driving capacity of the central city and the fact that most of them are engaged in local livestock and agricultural production, with their incomes largely unaffected by the pandemic. In addition, the low level of local daily consumption expenditure makes them more resilient to the pandemic.

#### 3.2.4. Urban Scattered Community

Compared with the two cities, the average resilience value of urban scattered communities in Lanzhou (0.4161) was slightly higher than that in Sarajevo (0.3831). Based on the economic resilience and social resilience, the two cities are basically similar, but in terms of infrastructural resilience and institutional resilience, Lanzhou is significantly higher than Sarajevo. While in terms of community capital resilience, Sarajevo’s urban scattered community is significantly higher than those of Lanzhou. Living in the Sarajevo’s scattered communities, residents have no obvious group differentiations in the education level, income level, society satisfaction level, and overall epidemic cognitive level. However, living in Lanzhou’s scattered communities, the education level, income level, as well as the overall epidemic cognitive level is relatively low. They are also less satisfied with the society, and there are obvious group differentiations among elderly groups, migrant workers, and ethnic minorities. There are also fewer activities in the community, including neighborhood mutual assistance and community cooperation, as well as a lack of common community memory. As a result, the urban scattered communities in Sarajevo are significantly higher than those in Lanzhou in social resilience and community capital resilience. In regard to infrastructural resilience, residents living in the urban scattered community in Lanzhou, similar to other community residents in the city, share the convenience of urban infrastructure such as supermarkets, public hospitals, civic parks, sports venues, and public transport. However, in Sarajevo, constrained by income levels and the ability to provide public services, scattered communities’ residents were confined to the vicinity of their neighborhoods during the pandemic. In terms of institutional resilience, the epidemic control efforts in Lanzhou were significantly higher than those in Sarajevo. In particular, the grid community management system made residents effectively included in the overall epidemic management of the city, and residents responded to the epidemic in a timely manner. Combined with the actual development of the two cities, it is reflected that there are some communities similar to “slums” in the city, which are scattered in the city, most of which are located around the city, some of which are located in the back streets of the main city. During the pandemic, these communities had low overall resilience values, and it was difficult for people living in these communities to recover from the impact of the pandemic.

## 4. Discussion

### 4.1. How to Build More Resilient Urban Communities

Through the above comparison research, we found the construction of an urban resilient community contains at least three aspects: material elements (the physical environment, and facilities), social environment (residents’ ideology, government organizations, governance and management), and main behavior body (government departments, community members, property managers, and residents, etc.). This study proposes how to build more resilient urban communities from the perspectives of government departments, community residents, and the physical environment of communities.

#### 4.1.1. The Government Department

(1) Emergency plan

The emergency plan can guide the community management department to carry out emergency prevention and control and work deployment. The emergency plan should fully absorb the opinions of the government, the residents, the experts, NGOs, and other powers, as well as take the epidemic perception, epidemiological investigation, medical treatment, policy consulting, material reserve, transportation logistics, improvement of facilities, perfecting information system, and so on into consideration, so as to form a complete set of community risk assessment, the epidemic monitoring, and emergency disposal plans.

(2) Urban planning

To formulate resilient city plans, we need to adhere to the principle of combining “let”, “prevent”, and “avoid”, improve the index system for resilient city planning, study and formulate special plans for resilient cities, and strengthen the rigid constraints on improving urban resilience in all territorial space plans. It is necessary to comprehensively measure community resilience through a combination of qualitative and quantitative methods. Special attention should be paid to community resilience in disaster and conflict environments, such as epidemic situations, including the layout of community medical institutions, the reservation of refuge places, the allocation of leisure space, and the distribution of community shops.

(3) Social mobilization

Given the sudden outbreak of large-scale urban disasters, such as the epidemic, it is difficult for a single city to bear the impact of the disaster on its own. It is necessary to mobilize the whole society to participate in epidemic prevention and control and community recovery. Cities, communities, and families should strengthen contact and interaction, support and assist each other, maintain communication and coordination, and take coordinated measures. We should also maintain a smooth flow between departments and between the higher and lower levels of the government, and strengthen the allocation of materials, information, and personnel.

(4) Urban perception system

Build a collaborative, comprehensive, sensitive and reliable urban sensing system. Realize the interconnection and real-time information sharing of all sensing systems through big data, the Internet of Things, and other means. We will improve the surveillance and early warning network for biosecurity and major infectious diseases, and coordinate and strengthen management of basic-level medical institutions, pharmacies, airports, railway stations, and other sentinel sites for epidemic prevention.

#### 4.1.2. Community Residents

(1) Adequate and Diversified Employment

Ensuring adequate employment is the most important means of improving the resilience of community residents. Having access to other sources of income to maintain a normal life is an important way to improve people’s economic resilience when livelihoods are affected by the pandemic. In the Internet era, more people should be encouraged to continuously improve their individual skills with which they make a living according to their interests, abilities, and majors. While doing their main job well, they should explore other business opportunities within their capacity and strengthen the diversity of their income sources.

(2) Savings and stable income

In the epidemic prevention and control stage, some regions adopted long-term home quarantine, working from home, and even curfews in emergency periods, which made some small and medium-sized enterprises, especially catering and entertainment service industries, difficult to operate. At the same time, workers in the industrial and service sectors are unemployed or between jobs, which had a certain impact on these people’s incomes. Occupational stability directly determines the income stability of residents. For residents with a single source of income, when the only economic source is blocked due to the pandemic, their economic resilience will inevitably decrease. Therefore, residents should have a sense of emergency, develop the habit of saving, and strengthen their skills and business capacity at the same time to find other ways to make a living when their career is hindered, so as to reduce the impact of the epidemic on their daily life.

(3) Proficient in Internet-use skills

In the Internet era, the spread of epidemic information is highly dependent on mobile phones and mobile Internet. For urban residents, the lack of information and communication ability affects their normal life to a large extent, bringing great inconvenience to travel, shopping, and other aspects, especially in public health emergencies. The government released the latest information about the epidemic through the Internet, and residents learned about the government’s requirements through the Internet. At the same time, residents should actively participate in community self-organization, such as communities, volunteer teams, and interest groups. Residents should participate in community collective activities, strengthen neighborhood interaction, and enhance community social network relations, so as to broaden the access to information and improve disaster response resilience.

#### 4.1.3. The Physical Environment of Communities

As the carrier of community response to disaster and emergency, the improvement of surrounding infrastructure construction level is an important way to enhance community resilience. The COVID-19 outbreak revealed that cities and communities still have many problems with the layout and configuration of infrastructure. The existing disaster response facilities are more focused on natural disasters and accidents, while the community protection and quarantine under public health emergencies are not enough, especially the facilities for emergency diagnosis and treatment resources, and supplies of living materials are still to be improved.

(1) Improve the primary medical care system

During the epidemic, the shortage of primary medical care resources led to a large number of people pouring into large hospitals, which strained resources and aggravated cross-infection. The hierarchical diagnosis and treatment system and two-way referral system can be used for reference to realize the orderly connection between community primary medical care and urban comprehensive medical care, so as to guarantee the medical needs of residents, reduce the pressure of large general hospitals and specialized hospitals, and promote the development of community hospitals at the same time.

(2) Improve public services and leisure space

We should make full use of urban reserved space to build temporary hospitals, and quickly convert some university gymnasiums, large venues, and hotels into makeshift hospitals or centralized quarantine points, which can effectively reduce the spread of virus and reduce cross infection. Therefore, it is recommended to reserve a certain scale of open space around the community as emergency standby sites, such as various kinds of squares, multifunctional community parks, parking lots, sports fields, and other outdoor sites, so as to provide residents with places for fitness exercise and leisure activities while preventing and controlling sudden disasters, and enhance the integration of residents into public space. The improvement of community public services and leisure space can also strengthen the improvement of environmental quality, reduce residents’ exposure to air pollution, and improve the living environment.

(3) Build a community life circle that combines daily life needs with response to epidemic

As the basic unit of urban spatial layout and epidemic prevention and control, the community should not only provide residents with residential, commercial and leisure services, public health, and other forms of support, but also meet the need of epidemic prevention and warning, verification and control, health monitoring, and mutual assistance and cooperation under the COVID-19 pandemic. During the epidemic prevention and control period, residents’ travel was restricted. A large number of public services and business places were closed, and residents’ daily travel was forced to be limited to the areas around the community to obtain daily supplies. In some extreme cases, the prevention and control measures, such as home quarantine, community closure, and building closure, made daily supplies for residents only available through online ordering, group purchasing, and community logistics procurement. The question of whether community surrounding service facilities can provide daily supplies for residents became prominent. Therefore, it is particularly important to build a 15 min community life circle that combines COVID-19 prevention and control and daily life needs. Life circle should contain the necessary first aid, medical treatment, comprehensive supermarket and material reserve, and other basic resources. At the same time, it should guarantee the community’s traffic, communication, and energy supply during the disaster prevention and control, so as to build a multi-level public service facilities system to narrow the residents’ activity range, and at the same time, meet the demand of residents in order to avoid the spread of the COVID-19 caused by a wide range of social life, striving to form an efficient and convenient community life circle.

(4) Improve the accessibility of the transportation and logistics system

In the event of a public health emergency, it is necessary to rely on the unified coordination and support dispatch at the regional and national levels to solve the shortage of emergency manpower and material resources in the affected areas. As the basis of emergency rescue, material transportation, and prevention and control layout, transportation facilities become the key to ensuring the safe flow of personnel and materials among different regions. Therefore, in order to ensure the safe emergency personnel flow of life, medical supplies, and so on, it is suggested to optimize the connection between the urban and regional transportation infrastructure, strengthen cohesion in the transport plane line redundancy configuration, perfect the emergency disaster relief logistics safeguard mechanism, set up special emergency interface and logistics channels, and meet the emergency relief supplies and personnel emergency deployment of timeliness requirements, so as to make transportation facilities more resilient.

### 4.2. Reasons for Differences of Eastern and Western Cities

Comparing Lanzhou and Sarajevo, we can figure that the communities show different resilience in the face of COVID-19.

#### 4.2.1. Different Histories of Urban Development between the East and the West

First, the history of urban population development is different. Sarajevo is a city with a long history. Since the Ottoman Turkish era, a certain scale of community lived on the hillside, so the main residents lived in the mountain slope courtyard community, while a large number of urban populations in Lanzhou appeared after the founding of the People’s Republic of China, and the residents concentrated in the urban apartment community. Second, the history of urban economic development is different. Sarajevo has a strong industrial economic foundation in history, but it was hit hard in the war, and its residents have strong economic awareness, experience, and ability to resist risks. However, Lanzhou’s economy was developing rapidly in the recent 30 years. Residents’ high debt and high consumption are prominent, and their economic awareness and ability to resist risks are weak.

#### 4.2.2. Cultural Differences between Eastern and Western Communities

First, neighborhood cultures are different. In Oriental culture, communities, especially in suburban communities and mountain slope courtyard communities, there are more exchanges, help, and contacts with each other, and it is easy for community residents to establish a relationship of trust. In Western culture, there are clear boundaries between neighbors, and the privacy of residents’ family life is strong. Second, residents have different cultures. Oriental culture has a strong sense of obedience. They rarely question the government and can actively cooperate with the government to implement the requirements of epidemic prevention and control. In Western culture, residents have strong individual consciousness and independent thinking habits. They often question government policies and express their views through marches and demonstrations.

#### 4.2.3. Different Ways of Governance between Eastern and Western Governments

In the East, the Chinese government is an authoritative and efficient government, and the network of social governance is relatively sound. It can usually respond quickly at the first time when the epidemic breaks out and complete the information transmission from the state to individual residents. However, in Bosnia and Herzegovina, the special government organization structure and governance mode determine its low governance efficiency, so it is difficult for the government to establish an effective governance network from the state to the local and then to the community, and the response to public health emergencies is slow but democratic.

### 4.3. Limitation of This Study

Through a comparative study of community resilience in two Eastern and Western cities, Lanzhou and Sarajevo, this study found the differences in the response of different types of urban communities to the COVID-19 epidemic. However, there are still some shortcomings in the study, which are mainly reflected in the following aspects:

First, in order to reflect the differences between different urban communities, the study divides urban communities into apartment communities, mountain slope courtyard communities, suburban communities, and urban scattered communities. However, in practice, the boundary between different communities is not particularly strict. For example, in the suburban community, there are communities with the type of mountain slope courtyard, which are nested with each other, and the spatial boundary is not very obvious.

Secondly, limited to the research conditions, the number of different types of community and the general questionnaire quantity is relatively small, which makes the difference among different types of communities not significant, resulting in less universal and persuasive results. The commonalities and differences of the resilience of different types of urban community response to emergent public health events still need to take further discussion.

## 5. Conclusions

It became an unchangeable fact that human beings will coexist with coronavirus for a long time. The improvement of community resilience, such as self-organization, self-adaptation, and self-recovery, became an urgent focus of urban management and construction under the COVID-19 outbreak. Through the comparative study of two cities, Lanzhou in China and Sarajevo in Bosnia and Herzegovina, it was found that cities under different institutional, cultural, and environmental backgrounds showed different resilience in coping with the impact of the epidemic. Through the study, it was found that, due to various reasons, such as residents’ understanding of the epidemic, government organization, and management, and physical conditions of communities and neighborhoods, Lanzhou and Sarajevo had certain differences in resilience in coping with the COVID-19 epidemic.

(1) The importance of community capital resilience is highlighted. Facing COVID-19, both cities showed that community capital resilience, which is based on shared values and urban spirit and characterized by mutual understanding, identity, help, and cooperation, is one of the most important resources for humanity to cope with the influence of COVID-19. In particular, community capital resilience became an important aspect to improve the overall resilience of the community, especially in the mountain slope courtyard community and the suburban community where the government public management is weak. On the one hand, in the main urban areas of both cities, community capital resilience value is lower in “modern” neighborhoods that are new and separate from where people live and work, but higher in “older” neighborhoods that were built earlier and where people lived for generations. On the other hand, the community capital resilience of residents in the suburbs of the cities is higher than that of main urban areas of the cities. Urban administrators should pay more attention to the construction of urban communities linked by blood and business relationships, encourage parents and children to live nearby, encourage employees to live near the company, and build urban communities with common community memories and stronger cohesion.

(2) There is insufficiency in urban infrastructural resilience. Although there is a significant difference in the level of infrastructure construction in Lanzhou and Sarajevo, the areas of high infrastructural resilience value in the two cities are highly concentrated along the main rivers. Residents in the suburbs and mountain slope gathering communities have a significantly lower perceived infrastructural resilience, which reflects the lack of infrastructural resilience in the cities, when facing the impact of the epidemic. In Lanzhou, due to strict epidemic control policies, it is difficult for relatively good urban infrastructure to play its due role. Forming effective connections between community residents and convenience stores, supermarkets, hospitals, and public transport systems, so as to make the urban infrastructure play its due role in the pandemic, became an urgent task to improve the urban infrastructural resilience. In Sarajevo, the lack of essential medical care, shopping places, and emergency shelter in suburban communities makes infrastructural resilience an important constraint on the overall improvement of the resilience of the community. At the same time, due to the poor public transportation infrastructure in Sarajevo, it is difficult to establish a fast and efficient logistics system for epidemic prevention and control.

(3) Ethnic characteristic and economic resilience are closely linked. In both cities, the economic resilience of communities is closely linked to the characteristics of the people who live in them. Those residents living in Sarajevo’s mountain slope courtyard area community and Lanzhou city’s urban apartment community mostly belong to the middle class and the class above. Their ability to resist economic risk in the outbreak is relatively strong. Whereas those who live in suburban communities, urban scattered communities, due to the lack of stable employment and stable income sources, are often at risk of losing their jobs during the pandemic, with poorer economic resilience. Sarajevo and Lanzhou, in particular, are home to a large number of “migrant workers” who just moved from the surrounding countryside to live in the city. They have no permanent residence in the city, no stable job, and no access to enjoy the social security of the city. They live in neighborhoods that are among the most vulnerable to economic resilience in the city.

(4) There is tremendous difference in institutional resilience. The Chinese government’s strong control over the epidemic was also fully demonstrated in Lanzhou. In Lanzhou, the government can efficiently make social mobilization on the various types of community, such as organizing rounds of free nucleic acid detection for all people, a universal vaccination, using the public health resources to perform concentrated treatment to reduce mortality in infected patients, implementing effective immobilized measures, and the regional dynamic reset for COVID-19 patients. Different types of communities in Lanzhou can basically implement consistent epidemic prevention and control policies, and there are few cases of inconsistent policies and inadequate management in different communities. However, in Sarajevo, the government is unable to provide effective assistance to COVID-19 patients or provide nucleic acid detection for the whole population, and the limited medical resources are only available for the critically ill patients and those who seek medical care voluntarily. The government imposed curfews during the pandemic, but it is unable to ensure that the curfew is strictly observed in urban scattered and suburban communities.

## Figures and Tables

**Table 1:** Measures of urban community resilience under the COVID-19 pandemic.

Target	Dimensions	Indicators	Elements	Weight of Elements	Indicator Property
Community Resilience	Social resilience (S)	Census register (urban/rural)	S1 Census register	0.0669	+
		Age of primary family members	S2 Age structure	0.1316	+
		Knowledge level	S3 Education degree	0.0387	+
		Attractiveness of the city	S4 Duration of residence in the city	0.0932	+
		Health condition	S5 Health condition	0.0723	+
		Information technology skill level	S6 The level of smartphone use	0.0569	+
		Epidemic prevention knowledge Reserve	S7 Understanding of epidemic prevention and control knowledge	0.0473	+
		Overall impact of the epidemic	S8 The impact of the epidemic on lives	0.0963	+
		Social satisfaction	S9 Satisfaction with the medical insurance system	0.0783	+
			S10 Satisfaction with pension insurance	0.0943	+
		Social information transfer	S11 Access to information related to the epidemic	0.1108	+
		Public health cognition	S12 Cognition of epidemic	0.1134	+
	Economic resilience (E)	Quarantine	E1 Have ever been in quarantine	0.0318	+
		Income	E2 Have or do not have income	0.0411	+
			E3 Income stability	0.0422	+
			E4 Category of Income source	0.0686	+
			E5 Impact of the epidemic on income	0.1087	−
		Occupation	E6 Whether your job have changed since the outbreak	0.0367	+
			E7 Nature of workplace	0.1047	+
		House asset	E8 Housing property	0.0737	+
			E9 Housing area	0.1185	+
		Food’s price	E10 Rising food’s price	0.0394	+
		Income-expenditure comparison	E11 Have you been living beyond your income during the epidemic	0.0339	+
		Hunger problem	E12 Impact of the epidemic on eating problem	0.1173	−
		Traffic problem	E13 Impact of the epidemic on traffic problem	0.0736	−
		Residence problem	E14 Impact of the epidemic on residence problem	0.1098	−
Community Resilience	Institutional resilience (G)	Emergency support	G1 Is there a emergency plan	0.3197	+
			G2 Whether the emergency manpower and material resources are sufficient	0.3410	+
		Information transfer	G3 Emergency epidemic information transfer channel	0.0635	+
		Public health knowledge spread	G4 Whether the community carries out disaster prevention training such as epidemic disease	0.0306	+
		Performance of Public Institutions	G5 Government’s organization ability and leadership	0.0557	+
			G6 Improvement of community service project after outbreak	0.0607	+
			G7 Performance of local governments in the epidemic	0.0491	+
			G8 Will an official be dismissed from office in the event of an apparent epidemic	0.0214	+
		Government executive ability	G9 Implementation of government’s grounded measures such as curfews	0.0266	+
		Epidemic prevention treatment	G10 The government’s measures for people infected with COVID-19	0.0131	+
			G11 The government’s measures for people possibly infected with COVID-19	0.0186	+
	Infrastructural resilience (I)	Medical service	I1 Community convenience level of medical service	0.1260	+
		Electricity service	I2 Community electricity power supply	0.0405	+
		Water supply service	I3 Community water supply	0.0416	+
		Internet service	I4 Community Internet	0.0475	+
		Traffic service	I5 Community traffic	0.1127	+
		Medical facility	I6 The number of medical facilities around the community	0.0914	+
		Entertainment facility	I7 The number of entertainment facilities around the community	0.1671	+
		Shopping facility	I8 The number of shopping sites around the community	0.0693	+
		Traffic convenience level	I9 The number of traffic stops around the community	0.0896	+
		Emergency placement site	I10 Temporary residence	0.1527	+
		Landscape planting	I11 Landscape planting	0.0615	+
	Community capital resilience (C)	Community participation	C1 Willingness to participate in community activities	0.1327	+
		Identity	C2 Community attachment/identity and sense of belonging	0.2860	+
		Social network relationship	C3 Neighborhood relationship	0.1055	+
		Community support	C4 The degree of community members’ willingness to help others	0.1326	+
		Community cooperation	C5 Community stakeholder cooperation	0.1271	+
		Community benefits	C6 Help from people around you	0.1176	+
		Shared history	C7 Collective memory	0.0983	+

## Data Availability

The data set is provided by Geospatial Data Cloud site, Computer Network Information Center, Chinese Academy of Sciences. (http://www.gscloud.cn).

## Appendix A

**Table A1:** Case communities in Lanzhou.

Community Type	Chengguan District	Qilihe District	Anning District	Xigu District
Urban apartment community	Luduhuayuan Lanzhou University Second Hospital’s family member residence Yantan’anju neighborhood Yunxiang neighborhood	Lantongchang neighborhood Mingzhujiayuan State-owned assets property fourth neighborhood Ximaijiayuan	Science and Education Town Ruinanzijun Tianyoujiayuan Rong’anju neighborhood	Xigurenjia 504 company neighborhood Xinlehuayuan Hailiangxi’anhuafu
Mountain slope courtyard community	Caochang Street Fulongping Taoshuping Qingbaishishangping	Hualinping Chaijiahe Mijiaping Shenjialing	Jinsha village Hongyi village Anningbao Shajingyi	Xiaoping Ma’ershan village Moujiaping Ya’erwa
Suburban community	Sangyuanzi village Dalanggou village Qingshiwan village Yangjiawan village	Yuanjiawan Huazhaizi village Gangjiaying Shuimoyuan	Nanpoping Jiaojiazhuang village Zhujiajing village Xianshuigou village	Xinghutai village Gangzhen village Xiping village Anmen village
Urban scattered community	Fu Jiaxiang community Jin chengguan community Jiaojiawan community Songjiatan community	Wangjiabao new village 205 neighborhood Yi’ningjiayuan Cuijiazhuang community	Wangjiazhuang community South gate community East street community Taolin community	Fuyuan small town Hongzeyuan Fengzeyuan Lintaojie community

**Table A2:** Case communities in Sarajevo.

Community Type	Centar Sarajevo	Novi Grad Sarajevo	Novo Sarajevo	Stari Grad Sarajevo
Urban apartment community	Breka; Ciglane Koševsko Brdo Marijin Dvor: Skenderija	Alipašino Polje Čengić Vila Dolac Dobrinja Otoka	Čengić Vila Gornji Velešići Grbavica Hrasno Kovačići Malta Pofalići	Baščaršija Bistrik Ferhadija Medrese
Mountain slope courtyard community	Betanija Mejtaš-Bjelave Pionirska Dolina	Alipašin Most Reljevo	Gornji Kovačići Hrasno Brdo Vraca	Bistrik Kovači Sedrenik Vratnik
Suburban community	Trebevicika Vranjace Sumarska Gornjo Vakafska	Rajlovac Svrakino Selo Hrasnica Reljevo	Hum Brdo Grahoviste Humska	Soukbunar Sirokaca Bistrik Vratnik
Urban scattered community	Ciglane Koševsko Brdo Rizaha Štetića	Humacka ploca Alipašin Most Porodice Šehić	Čengić Vila Braće Panjeta Reisa Fehima Spahe—čikma	Trcivode Mošćanica Zmajevac
